# Mandibular Dentoalveolar Expansion in Early Mixed Dentition Using the Clara Expander: A Case Series

**DOI:** 10.3390/children12070951

**Published:** 2025-07-18

**Authors:** Esther García-Miralles, Clara Guinot-Barona, Laura Marqués-Martínez, Juan Ignacio Aura-Tormos, Victor Marco-Cambra

**Affiliations:** 1 Faculty of Medicine and Health Sciences, Catholic University of Valencia, 46001 Valencia, Spain; esther.garcia@ucv.es (E.G.-M.); laura.marques@ucv.es (L.M.-M.); vmarccam@hotmail.com (V.M.-C.); 2 Faculty of Medicine and Dentistry, University of Valencia, 46010 Valencia, Spain; juan.aura@uv.es

## Abstract

**Objective**: Mandibular expansion remains controversial due to concerns about long-term stability and effectiveness. While maxillary expansion protocols are well established, investigations into mandibular expansion remain limited. This study evaluates the efficacy of the Clara Expander appliance for mandibular expansion in early mixed dentition, assessing skeletal and dental changes using cone-beam computed tomography (CBCT). **Materials and Methods**: This prospective longitudinal study was conducted in Valencia, Spain, with a population of healthy children aged 6–10 years presenting negative osseodental mandibular discrepancies. CBCT scans were performed before and after treatment to evaluate mandibular dimensional changes, with statistical analyses conducted and a significance threshold of *p* < 0.05. A total of seven subjects were included in this case series, allowing for a descriptive analysis of treatment outcomes within this specific clinical context. **Results**: CBCT analysis confirmed significant mandibular expansion following the Clara Expander protocol. Post-treatment findings showed statistically significant increases in dental parameters, including Tooth 6 (furcation, MD = −2.25; *p* = 0.015), Tooth E (furcation, cementoenamel junction, vestibular, lingual, all *p* < 0.001), Tooth D (all variables significant), and Tooth C (furcation, MD = −4.18; *p* = 0.002; cementoenamel junction, MD = −3.56; *p* = 0.015). **Conclusions**: The Clara Expander appliance effectively promotes skeletal and dental mandibular expansion, with minimal adverse effects. Its user-friendly, non-invasive design enhances patient compliance and outcomes, contributing valuable data to the field of mandibular expansion and informing future research and clinical applications.

## 1. Introduction

Mandibular deficiency, characterized by a narrow lower jaw relative to the upper jaw, is a common skeletal discrepancy in orthodontics and can lead to clinical manifestations such as dental crowding, malocclusion, and compromised airway function [1,2,3]. One therapeutic approach to address this deficiency is mandibular dentoalveolar expansion, which aims to increase the transverse dimension at the alveolar level, thereby creating additional space for the alignment of teeth and improving interarch relationships [4,5].

While rapid maxillary expansion is a well-established and thoroughly studied treatment modality for correcting transverse discrepancies in the upper arch [6,7], mandibular expansion remains a more controversial and less standardized procedure [2]. The controversy largely stems from anatomical differences: the maxilla contains a mid-palatal suture that permits skeletal expansion during growth [3,7], whereas the mandible is a single, continuous bone without a central suture [4]. As such, the potential for true skeletal expansion in the mandible is limited, and the changes observed following expansion are typically dentoalveolar in nature [5].

These anatomical constraints raise valid concerns regarding the stability and predictability of mandibular expansion. Excessive transverse forces may lead to undesirable side effects such as alveolar bone dehiscence, the buccal tipping of teeth, and post-treatment relapse, especially when the physiological limits of the bone are exceeded [5,6].

Moreover, evaluating transverse mandibular dimensions in the mixed dentition stage poses diagnostic challenges. Conventional two-dimensional imaging, such as panoramic radiographs, often fails to clearly delineate the alveolar bone due to the overlapping of primary tooth roots and unerupted permanent teeth. In this context, the Wala Ridge, defined as the anatomical junction between basal and alveolar bone, offers a stable reference point for assessing dentoalveolar expansion [7]. It can be used in both arches to quantify changes and guide treatment decisions.

Although maxillary skeletal expansion can sometimes trigger secondary dentoalveolar changes in the lower arch [8], appliances designed specifically for mandibular expansion may accelerate and optimize this response, particularly in growing patients. The use of such devices remains under-reported in the literature, and their effects—especially over the long term—require further clinical evaluation.

Therefore, the aim of this study was to explore the dentoalveolar changes observed in the mandibular arch following treatment with a novel expansion appliance, the Clara Expander, in children during the early mixed dentition stage. Using cone-beam computed tomography (CBCT), this case series seeks to assess the short- and mid-term transverse outcomes of this treatment protocol and contribute preliminary data to an area where clinical evidence remains limited. This study represents the first formal clinical evaluation and detailed description of the Clara Expander appliance published in the scientific literature.

**Figure 1:**
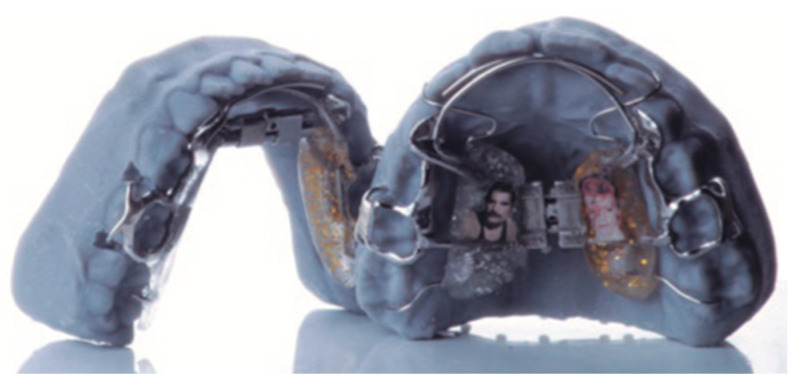
Clara Expander lower and upper appliances.

*The Clara Expander: Appliance Design and Features*. The Clara Expander is a custom-designed orthodontic appliance developed for mandibular dentoalveolar expansion in growing patients. Its structure is manufactured using a sintered metal process, providing high mechanical strength and reducing the risk of fracture during function. In the event of mechanical failure, the design allows for easy repair by replacing accessory wires, thereby increasing its clinical longevity and cost-effectiveness [9,10,11,12] (see Figure 1).

A distinguishing feature of the Clara Expander is the incorporation of Crozat-style clasps, which provide secure anchorage to molar teeth. These clasps, typically fabricated from high-strength stainless steel, are designed to engage the buccal and undercut areas of the teeth. Their “U”-shaped configuration allows for adaptation to slight anatomical variations in tooth position, while the occlusal rest component enhances vertical stability by preventing gingival migration during treatment [13,14,15,16,17,18].

The appliance is available in both removable and fixed configurations, enabling clinical flexibility based on the patient’s cooperation and treatment needs. A removable version can be converted into a fixed appliance using dental cement without requiring full appliance replacement, an option particularly useful in cases of limited compliance.

The lower framework of the Clara Expander is anatomically contoured to avoid direct pressure on the lingual frenum and minimize gingival contact. This design reduces the need for additional acrylic coverage in sensitive areas, potentially improving patient adaptation and oral hygiene maintenance.

In addition to its role in transverse dentoalveolar expansion, the Clara Expander can be modified with acrylic or resin components to serve as a mandibular advancement appliance, promoting forward positioning of the lower jaw. Although this additional functionality expands its potential clinical applications, further studies are required to evaluate its effectiveness in functional orthopedic protocols.

Compared to traditional appliances such as the Hawley or Schwartz, which rely on simple retention clasps that often lose adaptation and provide limited anchorage, the Clara Expander offers enhanced biomechanical performance through its Crozat-style clasps [10]. These ensure stable engagement and effective force transmission. Unlike the classic Crozat appliance—which benefits from similar clasp design but lacks an expansion screw—the Clara Expander combines controlled activation via a central expansion screw with elastic catenaries [13,14]. This synergy allows for efficient and stable mandibular dentoalveolar expansion in growing patients.

## 2. Materials and Methods

*Study Design.* An observational analytical study was conducted in accordance with the ethical principles outlined in the Declaration of Helsinki. Ethical approval was obtained from the Ethics Committee of the Universidad Católica de Valencia (UCV/2021-2022/185). The imaging protocol, included in this approval, was designed to balance scientific validity with the highest standards of radiological protection, particularly given the pediatric population involved.

Informed consent was obtained from the legal guardians of all participants following a comprehensive explanation of the study’s objectives and procedures. Each guardian received a copy of the informed consent document, who were informed of the potential risks and benefits of CBCT acquisition. Participants were also assured that their involvement was voluntary and that they could withdraw from the study at any time without any consequences to their clinical care.

To ensure participant confidentiality, data were collected using a unique identifier, along with gender and age. All study-related records were securely maintained, and participant information remained strictly confidential and inaccessible to third parties.

*Study Population.* The study population included children seeking orthodontic treatment at a private dental clinic in Valencia according to the following inclusion criteria: children aged 6 to 10 years, in the early mixed dentition phase, were included if they presented clinical signs of reduced transverse dimension in the mandibular arch—such as dental crowding, lingually displaced mandibular teeth, or crossbite—without prior orthodontic treatment. All participants were otherwise healthy, with no diagnosed syndromes or systemic conditions affecting oral or craniofacial development. Exclusion criteria included children with preexisting orthodontic appliances or space maintainers, those exhibiting non-cooperative behavior preventing accurate measurement and data collection, and individuals who failed to adhere to orthodontic follow-up appointments or prematurely terminated treatment.

*Use of CBCT:* Protocol, parameters, and ethical justification. Cone-beam computed tomography (CBCT) was employed in this study to accurately evaluate both dental and dentoalveolar skeletal changes in the lower arch, which cannot be reliably assessed through conventional two-dimensional imaging. Given the aim of the study—to analyze structural changes at the dentoalveolar level—three-dimensional imaging was considered essential to obtain reproducible and clinically relevant measurements, particularly in the transverse dimension.

CBCT scans were obtained at two time points: prior to treatment initiation and at the end of the expansion protocol, after a two-year follow-up period. The indication for CBCT was established only after a complete clinical diagnosis had been made using standard orthodontic records, including intraoral and extraoral photographs, study models, and panoramic radiographs. CBCT was not used for initial diagnosis but specifically for treatment planning and outcome assessment within the context of this prospective clinical study.

All scans were obtained using a Planmeca ProMax 3D Mid unit (Planmeca Oy, Helsinki, Finland) with an 8 × 8 cm field of view, 200 µm voxel size, and individually adjusted exposure settings, following ALADA principles. Child-specific protocols were applied, including pulsed exposure and automatic dose reduction. Patients were scanned while seated, with the head in a natural position standardized using the Frankfort horizontal and midsagittal planes. DICOM data were processed with Dolphin Imaging 11.9, and all measurements were performed by calibrated examiners following a predefined protocol to ensure consistency and reproducibility.

*Outcome Variables.* Different variables were included to be evaluated in the study, based on patients’ characteristics, on dental positions and on skeletal changes (see Table 1). To determine changes in the mandible, the Wala Ridge line was selected. It is defined as the junction between basal bone and alveolar bone, corresponding clinically to the mucogingival line. Wala’s analysis has been used as a diagnostic reference to determine the amount of expansion achieved at the end of orthodontic treatment [16,17].

*Intervention Workflow.* After confirming eligibility, orthodontic records—including photographs, CBCT scans, and study models—were collected to assess transverse discrepancies. Following active treatment with the Clara Expander, patients were monitored for two years without additional interventions. Final records were taken at the end of this period to evaluate both immediate outcomes and long-term stability.

Given the study’s longitudinal design and lack of a control group, natural growth effects cannot be ruled out; findings reflect both treatment and physiological development. Dentoalveolar and skeletal changes were assessed using initial and final CBCT scans. Two calibrated, experienced examiners performed all measurements. Calibration was conducted using five randomly selected CBCT scans measured independently by two trained examiners, repeated after a two-week interval. Inter- and intra-examiner reliability were assessed using the Intraclass Correlation Coefficient (ICC), yielding values ranging from 0.91 to 0.97 across all variables. These results indicate excellent measurement reproducibility

**Figure 2:**
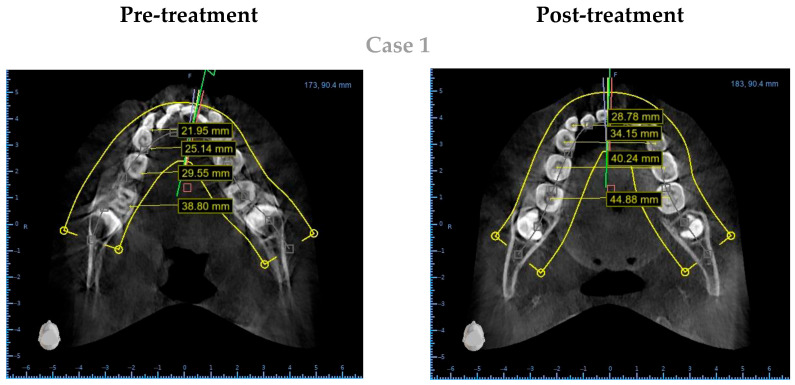
Example of pre- and post-treatment measurements on a patient sample.

To reduce bias, measurements were conducted blindly, with examiners unaware of scan timepoints. Data were recorded independently and compared to ensure agreement. (Figure 2).

*Statistical Analysis.* Data were anonymized, coded, and analyzed using SPSS version 23.0. Descriptive statistics included means and standard deviations for quantitative variables, and frequencies and percentages for qualitative ones. Due to the small sample and exploratory design, inferential analysis focused on identifying trends. Pre- and post-treatment comparisons were made using the Wilcoxon signed-rank test. A *p*-value <0.05 was considered significant. No formal sample size calculation was performed, as this pilot case series aimed to explore preliminary effects of the Clara Expander, with sample size based on patient availability.

## 3. Results

A total of seven children (four girls, three boys; mean age 8.2 ± 1.1 years) were included in this case series. All patients presented with reduced transverse dimension of the mandibular arch and were in the early mixed dentition stage at the time of treatment.

The distribution of continuous variables was assessed using the Shapiro–Wilk test. Given that several variables did not meet the assumption of normality, non-parametric methods (Wilcoxon signed-rank tests) were employed to compare pre- and post-treatment measurements (Table 2). Sample sizes varied across some variables due to the natural absence of certain teeth in some patients. Additionally, Wala Ridge measurements were limited to Tooth 6, as it was the only site where both right and left basal landmarks were consistently identifiable across all cases.

*Results of hypothesis testing for related samples.* The Wilcoxon signed-rank tests revealed statistically significant increases in several dentoalveolar dimensions following treatment with the Clara Expander, indicating measurable transverse changes across different segments of the mandibular arch.

For the permanent first molars (Tooth 6), a significant increase was detected at the furcation level (MD = −2.25 mm; *p* = 0.015), whereas differences at the cemento-enamel junction (CEJ), buccal and lingual surfaces, and dental position were not statistically significant.

In contrast, second primary molars (Tooth E) demonstrated significant post-treatment expansion across multiple parameters, including furcation (MD = −4.45 mm; *p* < 0.001), CEJ (MD = −3.58 mm; *p* = 0.001), buccal surface (MD = −6.05 mm; *p* < 0.001), and lingual surface (MD = −4.18 mm; *p* < 0.001).

Similarly, the first primary molars (Tooth D) showed statistically significant increases in all measured dimensions: furcation (MD = −4.55 mm), CEJ (MD = −4.35 mm), buccal (MD = −5.22 mm), lingual (MD = −3.49 mm), and dental position (MD = −4.50 mm), all with *p*-values ≤ 0.011.

Finally, in the primary canines (Tooth C), significant increases were observed at the furcation (MD = −4.18 mm; *p* = 0.002) and CEJ (MD = −3.56 mm; *p* = 0.015) levels, suggesting favorable expansion in the anterior region of the arch as well.

These findings are visually summarized in Table 3, which shows the magnitude and direction of statistically significant pre- to post-treatment changes across tooth groups and anatomical reference points.

## 4. Discussion

This case series suggests that the Clara Expander may promote and maintain mandibular dentoalveolar expansion in growing patients, potentially reducing relapse and the need for further interventions. Despite promising outcomes, results should be interpreted cautiously due to the exploratory design and lack of a control group.

The use of CBCT enhanced methodological precision, and the two-year follow-up provided insight into the stability of changes over time. Although formal patient-reported outcomes were not collected, clinical impressions indicated good comfort and aesthetics, suggesting high appliance acceptability. Future studies with standardized assessments and larger, controlled samples are needed to validate these findings.

An important consideration in interpreting transverse changes is the distinction between buccal tipping of teeth and true skeletal or alveolar expansion. To address this, our methodology included specific landmarks such as the furcation and, particularly, the Wala Ridge line, which reflects the basal–alveolar junction and is less affected by tooth inclination. By incorporating this reference point, we aimed to minimize the influence of dental tipping and improve the reliability of assessing structural expansion. While angular or cross-sectional analyses were not performed, the consistent increases observed at the Wala Ridge level across patients suggest that the effects are not merely dental but include alveolar components.

Comparing our findings with previous research, Goldman et al. examined dentoalveolar mandibular expansion using a banded appliance, reporting significant dental expansion and changes in molar angulation but no significant skeletal modifications. In contrast, the present study on the Clara Expander revealed both dentoalveolar and dental changes, emphasizing the importance of CBCT evaluation in orthodontic outcomes [5].

Similarly, Ugolini et al. evaluated mandibular width changes following rapid maxillary expansion (RME), reporting significant short-term effects that lacked long-term stability. In contrast, this case series with the Clara Expander demonstrated sustained increases in mandibular dentoalveolar width after two years without additional interventions. While the lack of a control group limits attribution solely to the appliance, the results suggest that direct lower arch expansion may offer greater long-term stability. Further comparative studies are needed to explore differences in stability between treatment approaches [19].

Although some dimensional changes may appear small in absolute terms (measured in millimeters), they hold considerable clinical relevance in the context of early mixed dentition. In growing children, even modest transverse gains of 2–4 mm can relieve anterior crowding, improve arch coordination, and reduce the need for extractions or future orthodontic interventions. These outcomes support the functional and preventive value of the Clara Expander in early treatment protocols.

The use of CBCT in this study is supported by previous research, including work by Colino Gallardo et al. and Abate et al., who demonstrated its superiority over 2D imaging in evaluating maxillary and mandibular expansion. These studies highlight CBCT’s ability to accurately capture skeletal and dentoalveolar changes. In the present case series, three-dimensional imaging was crucial for detecting subtle mandibular width modifications and ensuring the reproducibility of linear measurements over two years. The high-resolution data significantly enhanced the validity of the findings and the interpretation of clinical outcomes [20,21].

Additionally, a study conducted by Miranda at the European University of Valencia evaluated mandibular expansion using the Stage 1 appliance from the Orthotropics protocol—a device with design characteristics similar to the Clara Expander, including the use of Crozat clasps. That study reported significant improvements across multiple variables in the lower arch, such as intermolar width, intercanine distance, E–E distance, palatal cusp positioning, and mucogingival junction displacement, as well as an observed decrease in Wala Ridge width and overall expansion potential. While the methodologies and sample characteristics differ, the results are consistent with those of the present case series, supporting the notion that appliances incorporating direct lower arch expansion with Crozat-based anchorage may produce clinically relevant dentoalveolar changes. Comparative studies between different appliance designs would help further delineate their respective biomechanical effects and long-term outcomes [22].

Although not a primary objective of the present study, clinical observation during follow-up visits revealed a generally positive patient perception regarding comfort and aesthetics of the Clara Expander appliance. While these impressions were not formally assessed through validated instruments, they may play a relevant role in treatment adherence and overall satisfaction. In some cases, missing data in specific tooth positions was due to physiological exfoliation or congenital absence, which limited the availability of certain measurements. Future studies should consider including standardized measures of patient-reported outcomes to further explore this dimension of clinical effectiveness.

This case series was exploratory in nature, involving a small sample without a control group, which limits the ability to draw causal conclusions or generalize the findings. The limited sample size—particularly in comparisons involving only five subjects—reduces statistical power and increases the potential for type I error, which must be considered when interpreting the results. Although the two-year follow-up provides insight into post-treatment stability, the influence of natural craniofacial growth cannot be excluded, making it difficult to isolate the effect of the appliance. However, the consistency and directionality of the dimensional changes observed—especially at the Wala Ridge level—suggest a meaningful contribution of the Clara Expander to the outcomes obtained. The primary objective of this study was to describe treatment-related changes and generate preliminary data, rather than to perform definitive hypothesis testing. Additionally, the absence of standardized patient-reported outcome measures restricts the evaluation of patient-centered aspects. Future research with larger, controlled samples and growth-adjusted designs is needed to confirm these preliminary results and incorporate a systematic assessment of patient experiences, including the design of prospective randomized clinical trials (RCTs) to evaluate treatment effectiveness under standardized protocols

## 5. Conclusions

Within the limitations of this case series, the Clara Expander appliance may contribute to mandibular dentoalveolar expansion in growing patients, with outcomes that appeared stable over a two-year follow-up period. These preliminary findings suggest potential benefits, which should be confirmed with further investigation through controlled trials with larger samples and standardized outcome measures.

## Figures and Tables

**Table 1:** Study variables and measurement phases.

Category	Variable	Variable Type		Measurement Phase
Based on patients’ characteristics	Patient gender	qualitative (male/female)		pre-treatment
	Patient age	quantitative (months)		pre-treatment
Based on dental position	6-6 width	quantitative (millimeters)	width between mesial buccal cusps of permanent first molars	pre- and post-treatment
	E-E width	quantitative (millimeters)	width between mesial buccal cusps of second primary molars	pre- and post-treatment
	D-D width	quantitative (millimeters)	width between mesial buccal cusps of first primary molars	pre- and post-treatment
	C-C width	quantitative (millimeters)	width between cusps of primary canines	pre- and post-treatment
Based on dentoalveolar changes	6-6 Wala Ridge width	quantitative (millimeters)	width between the basal–alveolar bone junctions of the right and left permanent first molars, reflecting skeletal expansion in the posterior mandible	pre- and post-treatment
	E-E Wala Ridge width	quantitative (millimeters)	width the basal–alveolar bone junctions of the right and left second primary molars, assessing expansion in the middle mandibular segment	pre- and post-treatment
	D-D Wala Ridge width	quantitative (millimeters)	width between the basal–alveolar bone junctions of the right and left first primary molars, indicating skeletal width changes in the middle-anterior position	pre- and post-treatment
	C-C Wala Ridge width	quantitative (millimeters)	with between the basal–alveolar bone junctions of the right and left primary canines, evaluating the anterior segment of mandibular expansion	pre- and post-treatment

**Table 2:** Shapiro–Wilk tests of normality for study-dependent variables.

	Variable	Statistic (Shapiro–Wilk Test)	*p*
Pre-treatment	Furcation	0. 94	0.09
	CEJ	0.95	0.22
	Buccal	0.95	0.22
	Lingual	0.96	0.43
	Dental position	0.94	0.13
	Wala Ridge width 6 right	0.89	0.28
	Wala Ridge width 6 left	0.87	0.17
Post-treatment	Furcation	0.94	0.18
	CEJ	0.95	0.20
	Buccal	0.95	0.23
	Lingual	0.97	0.64
	Dental position	0.92	0.05
	Wala Ridge 6 right	0.90	0.35
	Wala Ridge 6 left	0.89	0.27

**Table 3:** Wilcoxon tests for paired samples for study variables based on pre- and post-treatment.

Tooth	Variables	Mean	N	SD	Paired Differences				
					MD	CI (95%) for MD		DF	*p*
						Lower	Upper		
6	Pre-furcation Post-furcation	45.57	7	1.74	−2.25	−3.89	−0.62	6	0.015
		47.82	7	2.05	-	-	-	-	-
	Pre-CEJ Post-CEJ	38.68	7	1.33	−1.23	−3.08	0.61	6	0.153
		39.91	7	1.88	-	-	-	-	-
	Pre-buccal Post-buccal	56.01	7	3.53	−2.94	−7.94	2.06	6	0.200
		58.94	7	3.33	-	-	-	-	-
	Pre-lingual Post-lingual	32.98	7	2.47	−2.98	−6.58	0.63	6	0.090
		35.96	7	2.08	-	-	-	-	-
	Pre-dental position Post-dental position	47.10	7	1.85	−1.04	−8.39	6.31	6	0.741
		48.14	7	6.50	-	-	-	-	-
	Pre-Wala ridge right Post-Wala ridge right	2.33	7	0.36	0.09	−0.13	0.30	6	0.361
		2.24	7	0.29	-	-	-	-	-
	Pre-Wala ridge left Post-Wala ridge left	2.26	7	0.34	0.09	−0.22	0.40	6	0.508
		2.17	7	0.23	-	-	-	-	-
E	Pre-furcation Post-furcation	36.88	5	1.19	−4.45	−3.31	−9.53	4	<0.001
		41.33	5	1.53	-	-	-	-	-
	Pre-CEJ Post-CEJ	31.30	5	1.67	−3.58	−2.25	−6.57	4	0.001
		34.88	5	2.01	-	-	-	-	-
	Pre-buccal Post-buccal	44.09	5	3.26	−6.05	−4.07	−7.47	4	<0.001
		50.14	5	2.60	-	-	-	-	-
	Pre-lingual Post-lingual	27.84	5	1.92	−4.18	−2.77	−7.26	4	<0.001
		32.03	5	1.11	-	-	-	-	-
	Pre-dental position Post-dental position	37.90	5	1.22	−3.55	0.16	−2.34	4	0.058
		41.45	5	4.37	-	-	-	-	-
D	Pre-furcation Post-furcation	29.68	5	1.26	−4.55	−3.05	−7.81	4	0.001
		34.23	5	0.86	-				
	Pre-CEJ Post-CEJ	25.45	5	0.99	−4.35	−2.79	−7.17	4	0.001
		29.80	5	1.06	-	-	-	-	-
	Pre-buccal Post-buccal	36.98	5	1.24	−5.22	−1.85	−3.98	4	0.011
		42.21	5	2.74	-	-	-	-	-
	Pre-lingual Post-lingual	23.59	5	1.24	−3.49	−1.26	−4.02	4	0.010
		27.08	5	2.07	-	-	-	-	-
	Pre-dental position Post-dental position	30.93	5	1.28	−4.50	−2.74	−6.57	4	0.001
		35.44	5	1.43	-	-	-	-	-
C	Pre-furcation Post-furcation	24.07	5	1.43	−4.18	−2.65	−7.59	4	0.002
		28.25	5	1.28					
	Pre-CEJ Post-CEJ	20.89	5	1.90	−3.56	−1.53	−4.86	4	0.015
		24.45	5	0.89	-	-	-	-	-
	Pre-buccal Post-buccal	29.62	5	1.21	−4.00	−0.92	−3.60	4	0.153
		33.62	5	1.69		-	-	-	-
	Pre-lingual Post-lingual	19.63	5	0.97	−3.14	−0.48	−3.28	4	0.200
		22.77	5	2.36	-	-	-	-	-
	Pre-dental position Post-dental position	25.63	5	1.18	−4.12	−1.74	−4.81	4	0.090
		29.75	5	1.35	-	-	-	-	-

NOTE: MD: mean difference; 95% CI MD: 95% confidence interval for the mean difference.

## Data Availability

The data supporting the findings of this study are available from the corresponding author upon reasonable request. Due to institutional policy, the datasets are not publicly available.

## References

[1] Rutili V., Nieri M., Franceschi D., Pierleoni F., Giuntini V., Franchi L. (2022). Comparison of rapid versus slow maxillary expansion on patient-reported outcome measures in growing patients: A systematic review and meta-analysis. Prog. Orthod..

[2] Rutili V., Mrakic G., Nieri M., Franceschi D., Pierleoni F., Giuntini V., Franchi L. (2021). Dento-skeletal effects produced by rapid versus slow maxillary expansion using fixed jackscrew expanders: A systematic review and meta-analysis. Eur. J. Orthod..

[3] Khan M.K., Sharma D.S., Jindal M.K. (2023). Unusual systemic and nondental effects of maxillary expansion therapy: A comprehensive and updated review of literature. J. Orthod. Sci..

[4] Ebrahimy G., Konermann A., El-Bialy T., Keilig L., Bourauel C. (2024). Comparative Experimental Evaluation of Orthodontic Appliances for Maxillary Arch Expansion. J. Clin. Med..

[5] Goldman R., Gandhi V., Malek F., Mehta S., Tadinada A., Goldman R., Yadav S. (2023). Skeletal and Dentoalveolar Changes with Mandibular Expansion in Growing Children. Cureus..

[6] Tamburrino R.K., Boucher N.S., Vanarsdall R.L., Secchi A. (2010). The transverse dimension: Diagnosis and relevance to functional occlusion. RWISO J..

[7] Motoyoshi M., Shirai S., Yano S., Nakanishi K., Shimizu N. (2005). Permissible limit for mandibular expansion. Eur. J. Orthod..

[8] Paoloni V., Giuntini V., Lione R., Nieri M., Barone V., Merlo M.M., Mazza F., Passaleva S., Cozza P., Franchi L. (2022). Comparison of the dento-skeletal effects produced by Leaf expander versus rapid maxillary expander in prepubertal patients: A two-center randomized controlled trial. Eur. J. Orthod..

[9] McGarry S., McNamara J.A., Franchi L.L., Ellis E. (2000). Mandibular expansion: A retrospective study of dentoalveolar effects. Am. J. Orthod. Dentofac. Orthop..

[10] O’Grady P., McNamara J., Baccetti T., Franchi L. (2006). A long-term evaluation of the mandibular Schwartz appliance and the acrylic splint expander in early mixed dentition patients. Am. J. Orthod. Dentofac. Orthop..

[11] Hang W. (2007). Obstructive sleep apnea: Dentistry’s unique role in longevity enhancement. J. Am. Orthod. Assoc..

[12] Mew J. (2007). Facial changes in identical twins treated by different orthodontic techniques. World J. Orthod..

[13] Kahn S. (2015). Let’s Face It!.

[14] Mew J. (1979). Biobloc Therapy. Am. J. Orthod..

[15] Mew J. (2010). Orthotropics: The Science of Dentofacial Balance.

[16] Gelb A. (2008). Myofunctional Therapy for Orofacial Dysfunctions.

[17] Crozat G. (1972). The Crozat System of Functional Orthodontics.

[18] Weaver K.E., Tremont T.J., Ngan P., Fields H., Dischinger T., Martin C., Richards M., Gunel E. (2012). Changes in dental and basal archforms with preformed and customized archwires during orthodontic treatment. Orthod. Waves.

[19] Ugolini A., Abate A., Donelli M., Gaffuri F., Bruni A., Maspero C., Lanteri V. (2024). Spontaneous mandibular dentoalveolar changes after rapid maxillary expansion (RME), slow maxillary expansion (SME), and leaf expander—A systematic review. Children.

[20] Colino P., Del Fresno I., Castillo L., Colino C., Baptista H., Criado L., Alvarado A. (2023). Skeletal and dentoalveolar changes in growing patients treated with rapid maxillary expansion measured in 3D cone-beam computed tomography. Biomedicines.

[21] Abate A., Ugolini A., Maspero C., Silvestrini F., Caprioglio A., Lanteri V. (2023). Comparison of the skeletal, dentoalveolar, and periodontal changes after Ni-Ti leaf spring expander and rapid maxillary expansion: A three-dimensional CBCT-based evaluation. Clin. Oral. Investig..

[22] Miranda Domínguez D.A. (2022). Predictibilidad de la Expansión Realizada por el Aparato Stage One en Dentición Mixta. Master’s Thesis.

